# Image Reconstruction Algorithm Based on Total Least Squares Target Correction for ECT

**DOI:** 10.1155/2021/3766877

**Published:** 2021-09-06

**Authors:** Lili Wang, Hexiang Lv, Deyun Chen, Hailu Yang, Mingyu Li

**Affiliations:** School of Computer Science and Technology, Harbin University of Science and Technology, Harbin, Heilongjiang 150080, China

## Abstract

In the image reconstruction of the electrical capacitance tomography (ECT) system, the application of the total least squares theory transforms the ill-posed problem into a nonlinear unconstrained minimization problem, which avoids calculating the matrix inversion. But in the iterative process of the coefficient matrix, the ill-posed problem is also produced. For the effect on the final image reconstruction accuracy of this problem, combined with the principle of the ECT system, the coefficient matrix is targeted and updated in the overall least squares iteration process. The new coefficient matrix is calculated, and then, the regularization matrix is corrected according to the adaptive targeting singular value, which can reduce the ill-posed effect. In this study, the total least squares iterative method is improved by introducing the mathematical model of EIV to deal with the errors in the measured capacitance data and coefficient matrix. The effect of noise interference on the measurement capacitance data is reduced, and finally, the high-quality reconstructed images are calculated iteratively.

## 1. Introduction

Electrical capacitance tomography (ECT) is a typical method for multiphase flow detection. The principle is to collect data through the electrode array installed on the outside of the pipe to make real-time visualization of the dielectric constant distribution inside the pipe, then to process, collect, filter, and amplify the capacitance data between the electrode pairs acquired by the sensor through the data acquisition unit, and then to reconstruct the image through the image reconstruction algorithm to carry out the image output to obtain the final image process. The ECT system has been gradually applied in the field of multiphase flow because of its noninvasive, fast response, simple structure, no radiation, wide range of applications, and good real-time performance [1–3]. The image reconstruction algorithm is the most critical step in the whole process of implementing capacitance tomography, which directly affects the clarity and accuracy of imaging, and is therefore a key point that needs to be addressed effectively.

The ECT system has a “soft field” effect, which makes the image reconstruction more difficult, and the obtained solution is only an approximate solution of the system, which has a certain error compared with the exact solution [4].Typical direct algorithms include the LBP algorithm, Tikhonov regularization algorithm, and so on [5]. The LBP algorithm is relatively simple, with fast imaging speed and low computation, but the disadvantage is that the reconstructed image is prone to distortion, blurred contours, and low accuracy, which is suitable for applications with low accuracy requirements. The Tikhonov algorithm has some advantages in dealing with pathology-related problems, but the solution of the algorithm is too smooth, resulting in a serious loss of information in the reconstructed image itself, so that the results are not satisfactory. The typical algorithm of the iterative class is the Landweber algorithm, which is simple in principle and has high image accuracy, but is not suitable for applications with high real-time requirements [6]. The improved Gauss–Newton image reconstruction algorithm and the Broyden family correction image reconstruction algorithm are recent image reconstruction algorithms, both of which have improved the reconstruction results to some extent [7, 8]. An optimized particle swarm combined with the Landweber algorithm for the case of getting stuck in a local optimum [9]. The gradient projection sparse reconstruction algorithm is used to address the problem of poor image accuracy when the medium is distributed close together in a two-phase flow [10].

To better solve the ill-posed nature of the inverse problem in the image reconstruction process and reduce the impact of complex noise on the reconstructed images, our work proposes an image reconstruction algorithm based on a combination of improved total least squares and EIV models, conducts simulation experiments on four flow types, and analyzes the impact of the noise environment on the imaging quality.

## 2. Basic Principles of Capacitance Chromatography Imaging

**Figure 1:**
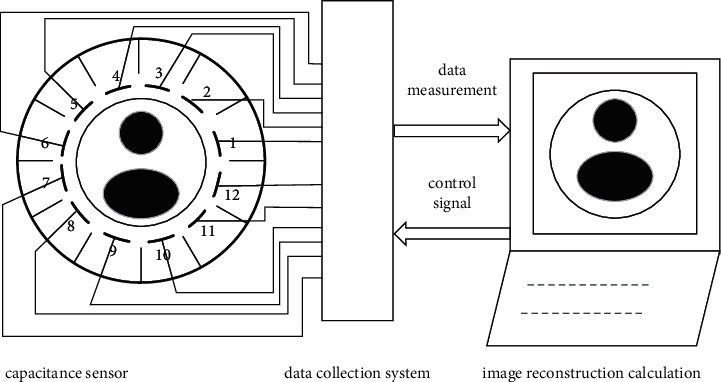
Structure of 12-electrode ECT system.

As shown in Figure 1, a 12-electrode ECT image reconstruction system consists of three parts: a capacitance sensor unit, a capacitance data acquisition and signal processing unit, and an image reconstruction unit. The capacitance sensor unit consists of an insulated pipe, measurement electrodes, and a grounded shield. The image reconstruction unit receives the capacitance data from the data acquisition unit, and it reconstructs images and calculates the other necessary parameters.

In the ECT system, the measured capacitance data between each electrode can be expressed as follows:

(1) $$\begin{array}{c} C_{i,j}=\int_{D}\int \varepsilon \left(x,y\right)S\left(x,y\right)\text{d}x\text{d}y. \end{array}$$

Among them, *C*_*i*,*j*_ is the capacitance between *i* and *j*, *D* is the cross-section to be measured, *ε*(*x*, *y*) is the distribution of relative permittivity in the region to be measured, and *S*(*x*, *y*) denotes the sensitivity distribution of the cross-section. The following mathematical model is obtained by normalizing the parameters:

(2) $$\begin{array}{c} C=SG. \end{array}$$

In equation (2), *C* is the *m* × 1 capacitance vector, *S* is the *m* × *n* sensitivity distribution matrix, and *G* is the *n* × 1 normalized dielectric constant distribution vector. When the dielectric constant changes, the capacitance data between different plates of the capacitance sensor change, and the capacitance data are sent to the data acquisition system, and then, the collected capacitance data and the known sensitivity field data are used to calculate the dielectric constant distribution in the pipe by the reconstruction algorithm, which is finally expressed as a visual image.

Image reconstruction is the inverse problem of the ECT system. The images are reconstructed by the measurement capacitance data, which are transfered from the data acquisition system according to dielectric constants of the flow. The image reconstruction can be defined as follows:

(3) $$\begin{array}{c} G=QC. \end{array}$$

In equation (3), *Q* is the *n* × *m* inverse sensitivity matrix. From equation (2), the inverse matrix exists only when *m* = *n*. Then, there are two problems in deriving equation (3) from equation (2):

1. Normally, the number of capacitance data measured is much smaller than the number of pixels of the reconstructed image, i.e., *m* < *n*; therefore, solving the inverse problem is solving a system of ill-conditioned equations, which is the typical ill-posed problem in ECT technology.
2. The inverse matrix of *S* does not exist, and equation (2) is a typical ill-conditioned equation whose solution is unstable; it means that when a perturbation is caused by the capacitor *C*, the gray scale *G* of the image changes along with it.

 The solution to the inverse problem of the ECT system is generally not available, and if it is, the solution is nonunique and unstable. This is the ill-posed problem of ECT image reconstruction.

 Because the required projection data are much larger than the actual projection data obtained, and because the sensitive field is influenced by the distribution of the medium in the object to be measured, errors are inevitable in the measurement process. Therefore, the impact of errors should also be taken into account during the calculation and image reconstruction.

 In this study, the total least squares theory is combined and applied to image reconstruction, which transforms the ill-posed problem into a nonlinear unconstrained minimization problem and avoids the matrix inversion problem.

## 3. ECT Image Reconstruction Based on Improved Ill-Posed Total Least Squares

### 3.1. Total Least Squares Theory

Least squares (LS) is ubiquitous in various applications that need to process observation data. The Gauss–Markov model (G–M model) is an adjustment model which takes into account the random error *e* of the observation vector *A* [11], and it is expressed as follows:

(4) $$\begin{array}{c} AX=b+e. \end{array}$$

Among them, *A* is the given *m* × *n* matrix, *b* is the m-dimensional known vector, *e* is the random observation error, and *X* is the variable to be estimated.

LS requires the input data matrix *A* to assume that there is no error, and all errors are limited to the observation vector *b*. Due to sampling error, human error, modeling error, and instrument error, the data matrix *A* may be inaccurate. Therefore, this assumption is usually unrealistic [12].

In this study, the total least squares theory is combined with the ECT image reconstruction algorithm model. *A* is the sensitivity matrix in the ECT system. The estimate *X* represents the result of the inversion of the capacitance and sensitive field data during the image reconstruction. Similarly, inaccuracies in data matrix *A* may be caused by various errors, which lead to biased results in the final image reconstruction.

In this case, the total least squares (TLS) method is designed. The concept of TLS was proposed in 1980 [13]. To fit the “best” subspace to the measurement data [*A*, *b*] means to seek the perturbation matrix *E* ∈ *R*^*m*×*n*^ and the perturbation vector *e* which can minimize ‖[*E*, *f*]‖_*F*_ and make equation (5) compatible, where ‖·‖_*F*_ is the Frobenius norm of the matrix [14, 15].

(5) $$\begin{array}{c} \left(A+E\right)X=b+e. \end{array}$$

### 3.2. Ill-Posed Total Least Squares Regularization Method

Based on the G–M model, the adjustment model and the least squares adjustment criterion are as follows:

(6) $$\begin{array}{c} \left\{\begin{array}{c} L=AX+e,e∼N\left(0,\sigma_{0}^{2}I\right),\\ f\left(e\right)=e^{T}e=\min. \end{array}\right. \end{array}$$

In equation (6), *A* is the *m* × *n* coefficient matrix, *L* is the *m* × 1 observation vector, *X* is the *n* × 1 unknown parameter vector, *σ*^2^ is the unit weight variance, and *e* is the *n* × 1 random error vector. The least square estimation and the covariance of the estimation are

(7) $$\begin{array}{c} \left\{\begin{array}{c} \hat{X}={\left(A^{T}A\right)}^{-1}A^{T}L,\\ \mathrm{cov}\left(\hat{X}\right)=\sigma_{0}^{2}{\left(A^{T}A\right)}^{-1}. \end{array}\right. \end{array}$$

The least squares estimator belongs to unbiased estimation, and the variance can be expressed as the trace of covariance matrix [16]:

(8) $$\begin{array}{c} D\left(\hat{X}\right)=\text{tr}\left[\mathrm{cov}\left(\hat{X}\right)\right]=\sigma_{0}^{2}\sum_{i=1}^{n}\frac{1}{\Lambda_{i}^{2}}. \end{array}$$

In the formula, Λ_*i*_ is the singular value of coefficient matrix *A*.

Considering the possibility of error in coefficient matrix *A*, the EIV observation model is introduced [17]; due to the complexity and diversity of measurement data, the adjustment of TLS in EIV model is reasonable. The EIV model is

(9) $$\begin{array}{c} L=\left(A+E_{A}\right)X+e. \end{array}$$

In equation (9), *L* is the *m* × 1 observation vector, *A* is the given *m* × *n* matrix, *E*_*A*_ is the error matrix of the coefficient matrix *A*, *X* is the *n* × 1 unknown parameter vector, and *e* is the random observation error.

Equation (9) can be expressed as follows:

(10) $$\begin{array}{c} \left\{\begin{array}{c} \begin{array}{c} L=\left(A+E_{A}\right)X+e=AX+\left[IX^{T}⊗I\right]\left[\begin{array}{c} e\\ e_{A} \end{array}\right],\\ e_{A}=\text{vec}\left(E_{A}\right) \end{array}\\ \left[\begin{array}{c} e\\ e_{A} \end{array}\right]∼N\left(\left[\begin{array}{c} 0\\ 0 \end{array}\right],\sigma_{0}^{2}\left[\begin{array}{cc} I_{n} & 0\\ 0 & I_{m}⊗I_{m} \end{array}\right]\right). \end{array}\right. \end{array}$$

In equation (10), *E*_*A*_ is the error matrix of coefficient matrix *A*, ⊗ is the Kronecker product, vec(*·*) is the straightening transformation, *I*_*n*_ is the unit matrix. The adjustment criterion is

(11) $$\begin{array}{c} f\left(E_{A},e\right)=e_{A}^{T}e_{A}+e^{T}e=\min. \end{array}$$

The Lagrange objective function is constructed as follows:

(12) $$\begin{array}{c} F\left(E_{A},e\right)=e_{A}^{T}e_{A}+e^{T}e+2\lambda^{T}\left[L-e-AX-E_{A}X\right]. \end{array}$$

It can be concluded that the normal equation is as follows:

(13) $$\begin{array}{c} A^{T}A\hat{X}-A^{T}L=\hat{X}\frac{{\left(L-A\hat{X}\right)}^{T}\left(L-A\hat{X}\right)}{1+{\hat{X}}^{T}\hat{X}}. \end{array}$$

Let ${\hat{\mu }}^{\left(k\right)}={\left(L-A{\hat{X}}^{\left(k\right)}\right)}^{T}\left(L-A{\hat{X}}^{\left(k\right)}\right)/1+{\hat{X}}^{\left(k\right)T}{\hat{X}}^{\left(k\right)}$, and we can get the iterative formula:

(14) $$\begin{array}{c} {\hat{X}}^{\left(k+1\right)}={\left(A^{T}A\right)}^{-1}\left(A^{T}L+{\hat{X}}^{\left(k\right)}{\hat{\mu }}^{\left(k\right)}\right). \end{array}$$

The least square estimation can be used as the initial value of iteration. The iteration will stop when $\left\|{\hat{X}}^{\left(k+1\right)}-{\hat{X}}^{\left(k\right)}\right\|<\varepsilon$ (*k* is the number of iterations, and *ε* is the iteration threshold).

Considering the possible errors in the coefficient matrix, the inversion process of normal matrix *A*^*T*^*A* will become very unstable, and the mean square error is used as the basis of valuation, It can be seen from equation (8) that when Λ_*i*_ is close to zero, the variance will be very large, resulting in that the estimated parameters are not referential [18].

In the EIV adjustment model, the regularization method adds a stable functional to the TLS adjustment criterion:

(15) $$\begin{array}{c} f\left(E_{A},e\right)=\text{vec}{\left(E_{A}\right)}^{T}\text{vec}\left(E_{A}\right)+e^{T}e+\alpha X^{T}RX=\min. \end{array}$$

In the formula, *R* is the regularization matrix, and *α* is the regularization parameter greater than zero. Parameters are based on

(16) $$\begin{array}{c} {\hat{X}}^{\left(k+1\right)}={\left[A^{T}A+\alpha \left(1+{\hat{X}}^{\left(k\right)T}{\hat{X}}^{\left(k\right)}\right)R\right]}^{-1}\left(A^{T}L+{\hat{X}}^{\left(k\right)}{\hat{\mu }}^{\left(k\right)}\right). \end{array}$$

The regularization iteration is performed according to equation (16). The iteration will stop when ${\hat{X}}^{\left(k+1\right)}-{\hat{X}}^{\left(k\right)}<\varepsilon$.

It can be concluded from equation (16) that the inversion of normal matrix *A*^*T*^*A* will become stable after adding the corresponding stable functional, and the estimated parameters will be reliable.

### 3.3. Ill-Posed Total Least Squares Targeted Singular Value Correction

The targeting matrix is based on the composition of eigenvectors corresponding to smaller eigenvalues [19], and its structure is shown in the following equation:

(17) $$\begin{array}{c} \hat{R}=\sum_{i=j}^{n}G_{i}G_{i}^{T}. \end{array}$$

In equation (17), *G*_*i*_ is the eigenvector corresponding to the small singular value of the normal matrix *A*^*T*^*A*. *A* value is a small eigenvalue when the sum of the standard deviation components of the eigenvalue accounts for more than 95% of the standard deviation, such as

(18) $$\begin{array}{c} \sum_{i=j}^{n}\frac{1}{\Lambda_{i}}\geq 95\%\sum_{i=1}^{n}\frac{1}{\Lambda_{i}}. \end{array}$$

In equation (18), Λ_*i*_ is the eigenvalue of *A*^*T*^*A*. The matrix $\hat{R}$ only corrects small singular values, and it can reduce variance and avoid unnecessary deviation at the same time and make the valuation more reasonable.

Ill-posed problems exist in the total least squares iterative algorithm. In the process of total least squares iterative calculation applied to ECT, the coefficient matrix changes slightly, and the targeting matrix also changes, which leads to the unreliability and error of the final estimation. This means that the sensitivity matrix is constantly changing slightly as it participates in the iterative calculation process, which can ultimately result in large errors in the image reconstruction. Aiming at the change of target matrix, the following target singular value correction method is adopted [20].

According to the EIV model *L*=(*A*+*E*_*e*_)*X*+*e* and ill-conditioned TLS adjustment criterion [21],

(19) $$\begin{array}{c} f\left(E_{A},e\right)=\text{vec}{\left(E_{A}\right)}^{T}\text{vec}\left(E_{A}\right)+e^{T}e+\alpha X^{T}RX=\min. \end{array}$$

Construct the Lagrange objective function:

(20) $$\begin{array}{c} F\left(E_{A},e\right)=e_{A}^{T}e_{A}+e^{T}e+\alpha X^{T}RX+2\lambda^{T}\left[L-e-AX-E_{A}X\right]. \end{array}$$

The first-order partial derivative of a formula is

(21) $$\begin{array}{c} \left\{\begin{array}{c} \frac{\partial F}{\partial e}=2e^{T}-2\lambda^{T}=0,\\ \frac{\partial F}{\partial e}=2e+2\lambda^{T}\left({\hat{X}}^{T}⊗I_{n}\right)=0,\\ \frac{\partial F}{\partial e}=2\alpha {\hat{X}}^{T}R-2\lambda^{T}\left(A+E_{A}\right)=0. \end{array}\right. \end{array}$$

The equation that *e*=*λ* and *E*=*λX*^*T*^ can be obtained from equation (20), and substitute parameters into the EIV model (9):

(22) $$\begin{array}{c} L-A\hat{X}=E_{A}\hat{X}+e=\lambda \left({\hat{X}}^{T}\hat{X}+1\right). \end{array}$$

According to equation (22),

(23) $$\begin{array}{c} e=\lambda =\left(L-A\hat{X}\right){\left({\hat{X}}^{T}\hat{X}+1\right)}^{-1}. \end{array}$$

Therefore, we can figure out that

(24) $$\begin{array}{c} {\hat{E}}_{A}=\lambda \hat{X}=\left(L-A\hat{X}\right){\left(\hat{X}\hat{X}+1\right)}^{-1}{\hat{X}}^{T}. \end{array}$$

Then, the coefficient matrix $\hat{A}$ is reconstructed by *E*_*A*_:

(25) $$\begin{array}{c} \hat{A}=A+E_{A}. \end{array}$$

Then, the parameter $\hat{X}$ is solved by the regularization method:

(26) $$\begin{array}{c} \hat{X}={\left[{\left(A+E_{A}\right)}^{T}\left(A+E_{A}\right)+\alpha R\right]}^{-1}{\left(A+E_{A}\right)}^{T}L={\left({\hat{A}}^{T}\hat{A}+\alpha R\right)}^{-1}{\hat{A}}^{T}L. \end{array}$$

The iterative calculation is carried out according to equations (24)–(26). The iterative formula is as follows:

(27) $$\begin{array}{c} \left\{\begin{array}{c} {\hat{E}}_{A}^{\left(k\right)}=\left(L-A{\hat{X}}^{\left(k\right)}\right)\left({\hat{X}}^{\left(k\right)T}{\hat{X}}^{\left(k\right)}+1\right){\hat{X}}^{\left(k\right)T},\\ {\hat{X}}^{\left(k+1\right)}={\left({\hat{A}}^{\left(k\right)T}{\hat{A}}^{\left(k\right)}+\alpha R\right)}^{-1}{\hat{A}}^{\left(k\right)T}L. \end{array}\right. \end{array}$$

The iteration will stop when ${\hat{X}}^{\left(k+1\right)}-{\hat{X}}^{\left(k\right)}<\varepsilon$.

## 4. Algorithm Implementation Steps

### 4.1. Pretreatment

A specific sensitive field strength and noise factor of the surrounding environment is preset to verify the feasibility of the algorithm by comparing the imaging accuracy and clarity of the algorithm under different environments through the control variables method. The capacitance data come from four different flow types: core flow, laminar flow, circulation flow, and multidrop flow, and the corresponding medium distribution matrices collected by the capacitance sensor array in the noise-free case and in different noise environments are simultaneously normalized to obtain the measurement data.

### 4.2. Algorithm Implementation

(1) The normalized data are brought into the least square estimation to get the iterative initial value *G*, and the least square function is constructed as follows:
  (28) $$\begin{array}{c} f\left(G\right)=\left\|\text{SG}-C^{2}\right\|=\min. \end{array}$$
Get *G* which satisfies the minimum value of the function result, and ${\hat{X}}_{0}$ is used as the initial value of iteration for subsequent steps;
(2) The initial error matrix *E*_*A*_ of coefficient matrix is obtained by substituting ${\hat{X}}_{0}$ into equation (27)
(3) Take *E*_*A*_ into equation (25) to get the initial value $\hat{A}$ of the modified coefficient matrix
(4) According to the modified coefficient matrix $\hat{A}$, the corresponding normal matrix is obtained, and the initial value $\hat{R}$ of the targeting matrix is constructed according to equation (17);
(5) According to the targeting matrix $\hat{R}$, use the L-curve method to obtain the corresponding regularization parameter *α*
(6) Iterative operation according to equation (27). When ${\hat{X}}^{\left(k+1\right)}-{\hat{X}}^{\left(k\right)}<\varepsilon$, the iteration ends and the experimental target valuation is obtained. The smaller the value of *ε*, the smaller the error between the results of the reconstructed image and the real image, but more iterations are calculated.

## 5. Simulation and Experimental Results

### 5.1. Experiment Preparation

Based on the above theory, this study conducts simulation experiments to verify the effectiveness of the algorithm and the effect of coping with the inverse problem. For the four flow types, core flow, laminar flow, circulation flow, and multidrop flow, the experimental parameters are preprocessed and the simulation experiments are performed using Matlab with the ECT system. The ECT system is a 12-electrode system with a pipeline split into 900 units.

### 5.2. Experimental Results and Analysis

In order to verify the feasibility of this study's algorithm in ECT image reconstruction, two evaluation metrics are introduced: image error and correlation coefficient. The calculation formula is shown in equation (29) as well as equation (30), and the correlation coefficient can reflect the similarity between the reconstruction result and the actual prototype.

(29) $$\begin{array}{c} \text{error}=\frac{\hat{e}-e}{e}\times 100\%, \end{array}$$

(30) $$\begin{array}{c} \rho_{e\hat{e}}=\frac{\sum_{i=1}^{N}\left(\hat{e}-\tilde{e}\right)\left(e-\bar{e}\right)}{\sqrt{\sum_{i=1}^{N}{\left(\hat{e}-\tilde{e}\right)}^{2}}\sqrt{\sum_{i=1}^{N}{\left(\hat{e}-\tilde{e}\right)}^{2}}}. \end{array}$$

In equation (30), *e* denotes the distribution of the dielectric constant of the original image and represents the gray value of the image, the average of which is $\bar{e}$; $\hat{e}$ denotes the distribution of dielectric constants of the image obtained after image reconstruction using the algorithm, and the average gray value is noted as $\tilde{e}$. *N* is the dimension of *e* and $\hat{e}$. The relationship between the change in the resultant image of the image reconstruction and the original image can be derived from the calculation of equations (29) and (30). The value of error  indicates the error between the resultant image of the image reconstruction and the real image, and the value of $\rho_{e\hat{e}}$ indicates the similarity between the resultant image of the image reconstruction and the real image. The magnitude of the closeness between the result and the original image is positively correlated with the value of $\rho_{e\hat{e}}$ and negatively correlated with the value of $\rho_{e\hat{e}}$.

#### 5.2.1. Comparison of Noise-Free Reconstruction Results

**Figure 2:**
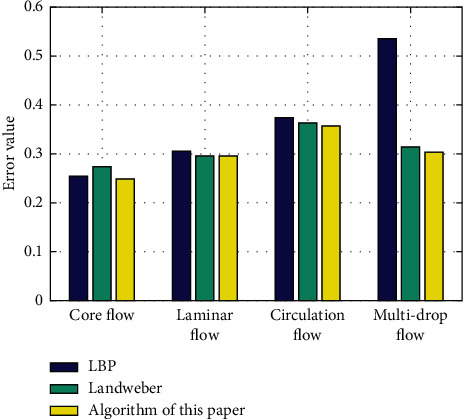
Error analysis without noise.

The image reconstruction process was simulated using the classical LBP algorithm, the classical Landweber algorithm, and the algorithm of this study for core, laminar, circulation, and multidrop flows, respectively. The imaging results were divided into 900 pixel units using a circular grid, and the reconstruction results without noise interference are given in Table 1, and the error comparison is given in Table 2 and Figure 2.

From the error results, it can be seen that the reconstruction accuracy of the algorithm in this study is improved to different degrees for the four selected flow types. Compared with the LBP algorithm, the reconstruction effect of the algorithm in this study is significantly improved in the case of loop flow and multidrop flow, and compared with the Landweber algorithm, the reconstruction effect is significantly improved in the case of core flow.

#### 5.2.2. Comparison of Noise-Free Reconstruction Results

In this study, the improved total least squares algorithm theory is selected for image reconstruction, with the aim of solving the problem that both the measurement data and the coefficient matrix have different degrees of error in a noisy environment. To verify the adaptability of the algorithm to noise, experiments are conducted for four flow types: core flow, laminar flow, circulation flow, and multidrop flow, and random noise is added to the normalized capacitance *C* and coefficient matrix *S*, respectively, *e*_*L*_ ~ *N*(0, *σ*^2^*I*_*m*_), *e*_*A*_ ~ *N*(0, *σ*^2^*I*_*m*_ ⊗ *I*_*n*_), *σ*=0.1, random noise generated by Matlab.

**Figure 3:**
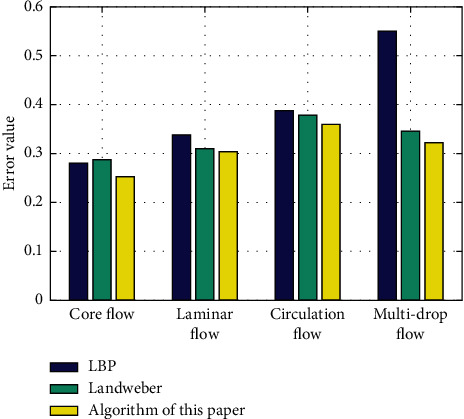
Error analysis with noise.

The LBP algorithm, Landweber algorithm, and the algorithm in this study were used for image reconstruction in this experimental setting. Due to the randomness of adding noise, to increase the general adaptability of the experiment, the validation data were tested 30 times, and finally, the image reconstruction was compared by calculating the average value, and the reconstruction results are given in Table 3, and the error comparison is given in Table 4 and Figure 3.

As can be seen from Figure 3, by adding noise, the overall error value of the algorithm in this study is low, and the image reconstruction accuracy and robustness are good, which have certain advantages compared with other algorithms; thus, it can be concluded that the algorithm in this study has good antiinterference ability under complex noise environment.

## 6. Conclusion

In this study, we propose an ECT image reconstruction algorithm based on the improved total least squares theory. Based on the analysis of the ill-posed nature of the total least squares problem iteration and the ill-posed nature of the ECT inverse problem, we construct adaptive targeting matrices based on the total least squares theory for the problem that both measurement data and coefficient matrices have different degrees of error in complex noisy environments and use the EIV model as the basic model for image reconstruction. The simulation results show that the resistance to different kinds of noise is effectively enhanced, which can better improve the situation that the image reconstruction process is easily distorted by noise, while improving the robustness and reconstruction accuracy of the reconstructed image, thus providing an effective method for ECT image reconstruction and providing a reference for subsequent research.

## Figures and Tables

**Table 1:** Reconstruction comparison without noise.

Flow type	Core flow	Laminar flow	Circulation flow	Multidrop flow
Original image	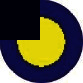	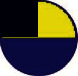	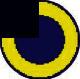	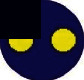
LBP algorithm	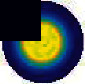	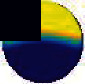	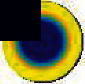	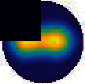
Landweber algorithm	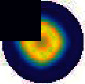	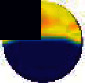	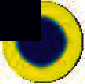	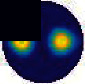
Algorithm of this study	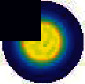	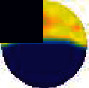	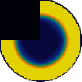	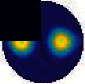

**Table 2:** Comparison of errors without noise.

Flow type	Core flow	Laminar flow	Circulation flow	Multidrop flow
LBP algorithm	0.25419	0.30449	0.37352	0.53718
Landweber algorithm	0.27336	0.29671	0.36381	0.31372
Algorithm of this study	0.24845	0.2965	0.35791	0.30335

**Table 3:** Comparison of reconstruction results with noise.

Flow type	Core flow	Laminar flow	Circulation flow	Multidrop flow
Original image	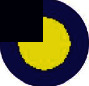	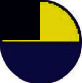	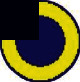	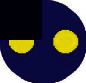
LBP algorithm	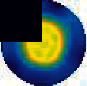	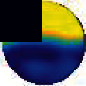	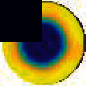	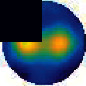
Landweber algorithm	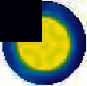	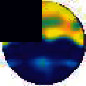	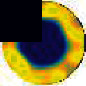	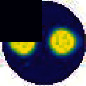
Algorithm of this study	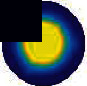	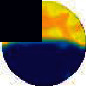	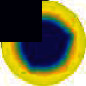	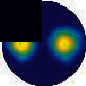

**Table 4:** Comparison of errors with noise.

Flow type	Core flow	Laminar flow	Circulation flow	Multidrop flow
LBP algorithm	0.27994	0.33879	0.38838	0.5612
Landweber algorithm	0.28809	0.31012	0.37915	0.34572
Algorithm of this study	0.25214	0.30354	0.36019	0.32267

## Data Availability

The data used to support the findings of this study are available from the corresponding author upon request.
